# Host immunity and vaccine development against Dengue virus

**DOI:** 10.1016/j.imj.2021.12.003

**Published:** 2022-02-06

**Authors:** Enhao Ma, Gong Cheng

**Affiliations:** a Tsinghua-Peking Center for Life Sciences, School of Medicine, Tsinghua University, Beijing, China; b Institute of Infectious Diseases, Shenzhen Bay Laboratory, Shenzhen, Guangdong, China; c Institute of Pathogenic Organisms, Shenzhen Center for Disease Control and Prevention, Shenzhen, Guangdong, China

**Keywords:** Dengue virus, Dengue vaccine, Cellular immunity, Humoral immunity, Efficacy

## Abstract

Dengue is one of the most prevalent and influential mosquito-borne viral infections in the world. According to 1 modeling estimate, the World Health Organization indicated that the annual cases of dengue virus (DENV) infection are as high as 390 million, placing more than half of the global population at risk. Despite its overwhelming prevalence, there is still no effective control method for containing this rapidly spreading disease. The only vaccine currently licensed for use is Dengvaxia, a tetravalent chimeric vaccine developed by Sanofi Pasteur in 2019. However, this vaccine fails to elicit a balanced immune response against all 4 serotypes of DENV and causes severe side effects in seronegative vaccine recipients. Vaccines naturally stimulate cellular and humoral immune responses, which are crucial for vaccine immunogenicity and clinical protection. This article focuses on these 2 immune responses triggered by DENV and their relationship with the efficacy of various vaccine candidates. This information will help us understand the vaccine immune response and relevant clinical protective efficacy further, providing insights into the development of new vaccines. Finally, we summarize feasible approaches for future vaccine development.

## Highlights

• Dengue virus and disease introduction.
• Dengue vaccine.
• Host immune response.
• Vaccine efficacy and future development insight.

## 1 Dengue virus

Dengue virus (DENV) is a single positive-stranded virus belonging to the genus *Flavivirus*. As a mosquito-borne virus, it mainly infects mammals through the vectors of female *Aedes aegypti* and *Aedes albopictus*. Other viruses belonging to *Flavivirus* with clinical importance include Yellow Fever virus (YFV), Zika virus (ZIKV), West Nile virus (WNV), and Japanese encephalitis virus (JEV), which all rely on mosquitoes in tropical or subtropical areas for transmission [1]. Therefore, the co-infection and co-transmission of mosquito-borne viruses and subsequent serious complications have become a new research direction.

In most cases, symptoms of DENV infections are similar to those of ordinary flu and rarely cause death. The World Health Organization (WHO) classifies the DENV infection into 2 main categories, a common self-limited illness, dengue fever (DF), and in rare cases, severe dengue (DHF/DSS) [2]. The former is accompanied by a 40 °C fever, with the possibility of severe headache, eye pain, muscle and joint pain, nausea, and vomiting [3]. The latter is classified into dengue hemorrhagic fever (DHF) and dengue shock syndrome (DSS). For DHF, patients’ blood platelet numbers fall to extremely low levels (<100,000/mm^3^) resulting in uncontrollable bleeding [4]. For DSS, the situation is more severe. Patients with the lethal syndrome have a pulse pressure below 20 mmHg (2.7 kPa) and develop various symptoms including “organ failure, hemorrhage fever, capillary leakage, shock” and finally, death [4], [5]. Unfortunately, there is a lack of specific antiviral drug treatments for severe dengue (DHF/DSS) although proper infusion treatment can effectively reduce the death rate from 20% to approximately 1% [3].

Dengue fever is mainly triggered by 4 antigenically distinct DENV types (DENV-1 to DENV-4) common in urban areas, each of which produces a different immune response [6]. In 2007, scientists isolated a novel fifth serotype from a patient in Sarawak, Malaysia, although this stereotype circulates in wild fields rather than urban areas [7]. The 4 serotypes of DENV share at least 65% genetic similarity, but each serotype can itself exhibit more than 6% variation [8]. Typically, infection with 1 serotype provides long-lasting homotypic protection but enhances the severity of subsequent heterotypic infections, making vaccine development very challenging [6,[9], [10]].

Therefore, despite concerted efforts over the past decades, only 1 dengue vaccine is currently approved for use, Dengvaxia, and its usage is limited. Dengvaxia is a live attenuated tetravalent chimeric vaccine that provides moderately effective protection against a single serotype, although the unbalanced immune response does not effectively protect vaccinators with prior infection [6].

### Structure of dengue virus

The dengue virion is a 50-nm enveloped particle surrounded by a capsid with icosahedral symmetry [11]. Like other flaviviruses, its genome contains 11,000 nucleotides and is between 9.4 kb and 13 kb in length. A single open reading frame in the genome is flanked by 5′ and 3′ untranslated regions (UTR) [12]. The opening frame encodes 3 structural proteins (capsid (C), precursor membrane (prM), and glycosylated envelope (E)) and 7 non-structural proteins (NS1, NS2A/B, NS3, NS4A/B, and NS5) [13]. Entering the late stage of infection, prM protein is cleaved into membrane protein (M) by furin protease, affecting the transmission capacity of the virus [14].

Three structural proteins have essential roles in the attachment, entry, assembly, and secretion process of viral infection. The nucleocapsid protein is responsible for wrapping the DENV genome and provides physical protection. The prM protein forms a complex with the commonly generated E protein, which helps stabilize its pH-sensitive epitopes [15]. The E protein, composed of 3 distinct domains (EDI, EDII, and EDIII), is the major surface protein and is essential for transmembrane fusion [96]. EDIII contains various neutralizing epitopes and is crucial for host cell surface binding [16], [17]. Because the E protein is involved in viral invasion, scientists regard it as an excellent vaccine target and antiviral drug candidate.

In addition to structural proteins, non-structural proteins (NPs) are essential for immune escape, viral enzymatic activity, and replication [18]. Among them, NS1, NS3, and NS5 are essential immunogenic proteins, which induce effective humoral or cellular immune responses against viral infection. NS1 is a glycoprotein lysed from the E protein and is mainly involved in RNA replication of the virus. The infected host cell secretes a soluble NS1 hexamer composed of 3 groups of homologous dimers, and because it accumulates in the serum, NS1 is used as a biomarker for the diagnosis of dengue fever [19], [20], [21]. In addition, NS1 protein acquires antigenic epitopes associated with type I and type II major histocompatibility complex (MHC), inducing a T cell immune response against the virus [22]. Recent studies have revealed that the monoclonal antibodies (mAb) 2B7 and 1 g 5.3 against NS1 effectively inhibited endodermal dysfunction and reduced viremia caused by DENV, ZIKV, and WNV [23], [24]. In these types of cases, NS1 has become the most promising non-structural protein target for vaccine candidates, capable of inducing cellular immunity and broad-spectrum humoral immunity.

## 2 Overview of dengue infection worldwide

DENV is a globally eminent pathogen that has increased public health concerns. Influenced by global climate change, international trade, frequent transnational activities, and accelerated urbanization, the number of WHO-reported dengue cases has increased strikingly eightfold during the last two decades [3]. The number has risen from 505,430 in 2000 to 4.2 million in 2019, and between 2000 and 2015, the death rate has increased 4-fold [3]. The endemic is most severe in developing and underdeveloped countries such as Brazil, the Philippines, Vietnam, Mexico, and Malaysia [25]. Among them, Brazil is the most severely affected, with all known DENV serotypes being identified [25]. In 2019, more than 100,000 cases were reported in these countries, and for Brazil, this number reached 2 million [25]. The situation has worsened because these countries are also breeding grounds for other arboviruses. A seropositivity study in Rio de Janeiro, Brazil, estimated that only 17% of the local population had not been infected with DENV, ZIKV, or Chikungunya virus (CHIKV), demonstrating the inefficacy of the current surveillance information systems to reflect actual cases of each infectious disease [26]. In Rio de Janeiro, the positive rate of ZIKV and CHIKV serum tests was estimated to be 5 times and 45 times higher than that of notified cases, respectively [26]. Because there is a tendency to have more dengue cases in underdeveloped and developing countries, the WHO defined the disease as a neglected tropical disease (NPD), indicating that the disease remains largely uncontrolled globally, despite its obvious severity [27].

Therefore, the implicit influence is much greater than the case numbers. Approximately 3.9 billion people are now living in areas prone to DENV infection [28], [29], which will lead to an immense economic burden in addition to the direct health impact [28,[30], [31]]. Dengue virus can cause a substantial economic burden in 4 main areas: the disease itself, surveillance and case reporting, prevention and control measures, and most importantly, post-outbreak management. Shepard et al. revealed that the annual economic loss in 2013 due to dengue fever was estimated to be $8.9 billion, equivalent to the total GDP of some small countries [32]. In addition, a survey in Taiwan comparing the economic costs (ie, inpatient, outpatient, emergency room, drugs, and loss of labor due to death) reported that the overall cost was 12.3 times higher in epidemic years than in non-epidemic years [33]. Therefore, mosquito-borne infectious viruses such as DENV can significantly impact the economy and people's livelihood, especially in the context of the coronavirus pandemic. Only by developing effective dengue prevention and control strategies as early as possible can countries reduce the heavy economic burden.

### 2.1 Transmission route of dengue virus

DENV has 2 transmission cycles: the urban cycle and forest cycle. For both cycles, mosquitoes (*A. aegypti and A. albopictus*) act as vectors that infect primate hosts. When female mosquitoes in urban areas obtain DENV via blood-sucking, the virus infects the midgut, and in about 8 to 12 days, the virions propagate and diffuse to the buccal cavity and salivary gland, where the virus is eventually spread to humans [34]. Similarly, non-human primates living in the forest are natural hosts for DENV infection. As the boundary between cities and the wild becomes closer, the 2 cycles can interact significantly impeding the prevention of disease.

### 2.2 Containment of dengue virus

The control and containment of disease are based on controlling the transmission vector, the mosquito. For instance, as a base camp for dengue fever, Singapore deploys environmental management instruments for DENV prevention. Because of the abundant rainfall and hot weather in Singapore, the local government applies strict law enforcement and periodic environment surveillance to eliminate any puddles or standing water, which promotes the mosquito life cycle. Singapore has also adopted an Israeli subspecies of *Bacillus thuringiensis* (Bti), which can kill mosquito larvae, as an environmentally friendly biological means to eliminate mosquito larvae in river channels and ponds [35]. Another commonly used biological measurement for vector containment is to infect the mosquitoes with Wolbachia bacteria, which blocks the virus by disrupting the normal function of the zygote or by passing the infection to the next generation via vertical transmission [36].

## 3 Development of a dengue vaccine

Preventing disease in advance is always preferable to treating it. Vaccines have long been considered as the most efficient strategy against disease, and thus continuous efforts have been made to develop an effective dengue vaccine since its first outbreak. Starting in the 1920s, 5 types of vaccines, including live attenuated, inactivated, recombinant subunit, viral vectored, and DNA/mRNA vaccines, have been developed, each with its own strength and weakness [37]. As of 2021, the first approved vaccine, Dengvaxia, has become commercially available, yet it has some problems [38]. In this review, we focus on 2 types of vaccines that are the most acceptable currently.

### 3.1 Live attenuated vaccines

Live attenuated vaccines contain live virus particles, which are attenuated to be less virulent or even avirulent [39]. Because of their resemblance to the virus, these vaccines can deliver a set of protective antigens that trigger a more robust immune response [40]. As 1 of the most traditional types of vaccine, live-attenuated vaccines are now the mainstream for dengue vaccine development, including 3 currently accepted vaccines: Dengvaxia, TAK-003, and TV003 [37].

Dengvaxia, developed by Sanofi Pasteur, is a live attenuated tetravalent vaccine developed using a chimeric approach. Another common name of this vaccine is CYD-TDV. To date, the vaccine has been licensed for use in more than 20 countries worldwide. Three doses of the vaccine are administered 6 months apart at 0, 6, and 12 months, ensuring an effective immune response in the long term [6]. The chimeric vaccine technique was developed at St. Louis University to construct a JEV vaccine candidate and was later adopted by Sanofi Pasteur [41], [42]. This vaccine uses the backbone of a 17D attenuated YFV strain (chimeric yellow fever dengue – CYD) that has been determined to be safe [6]. Using recombinant DNA techniques, the nonstructural gene of CYD was combined with the structural premembrane (prM) and envelope (E) genes of the 4 DENV serotypes to induce a DENV-specific immune response [6]. Notably, Dengvaxia lacks the nonstructural proteins NS3 and NS5, the main immunogenic proteins of DENV that induce cellular responses, and the specific CD4^+^ and CD8^+^ cellular immune responses induced by YFV have limited cross-reactivity [43]. Therefore, Dengvaxia mainly triggers humoral immune responses rather than cellular immune responses.

Another vaccine using the tetravalent live attenuated strategy is TV003, developed by the National Institute of Health (NIH) in the United States after the introduction of Dengvaxia. Compared with Dengvaxia that uses a chimeric approach based on the backbone of YFV, TV003 integrates monovalent vaccines with the live attenuated tetravalent vaccine (LATV) [44]. After studying prior vaccine strategies and problems of mutual interference and immune dominance, the NIH focused attention on the immunogenicity and safety of each monovalent antigen component. As a result, the vaccine has a balanced immune response against all 4 DENV serotypes [45], [46]. In the preliminary study on mice, macaques, and mosquitoes, the NIH tested the infectivity of several attenuated vaccine candidates and identified 8 monovalent vaccine components with the highest immunogenicity and safety [44]. After constituting these 8 monovalent vaccine components into 4 combinations (TV001–004), the NIH finally identified TV003 as the tetravalent vaccine that elicited the most balanced immune response, reaching 100%, 50%, 85%, and 100% positive serum antibodies for DENV1, −2 −3, and −4, respectively [47]. TV003 contains 4 attenuated recombinant dengue vaccine components, rDEN1D30, rDEN2/4D30, rDEN3D30/31, and rDEN4D30, which are the respective monovalent components of DENV1–4 [47]. For DENV1, −3, and −4, the candidate components are constructed by a ∼30 nucleotide deletion in the untranslated 3′-UTR region of the corresponding genome [48]. For DENV2, rDEN2/4D30 uses attenuated DENV4 as the backbone to express prM and E proteins, and it is the only chimeric vaccine component of the constructed vaccine. Because TV003 contains the complete backbone of 3 DENV serotypes, it can induce more comprehensive DENV-specific cellular responses compared with Dengvaxia and TAK-003 [49]. Another advantage of TV003 is that it can generate an antibody response in 90% of seronegative populations with only 1 vaccination dose, which would only be achieved by 3 doses of Dengvaxia or 2 doses of TAK-003 [50].

Another recombinant tetravalent vaccine candidate is TAK-003 (previously known as DENVax), which obtains its name from the Takeda pharmaceutical company [51]. Similarly, TAK-003 is also a live attenuated vaccine generated using a chimeric approach, yet unlike CYD-TDV, it uses an attenuated PDK-53 DENV2 strain as its genetic backbone and chimeric carrier [52]. PDK-53 is a laboratory-derived virus, and it is generated by passaging wild-type D2 16,681 virus through PDK cells 53 times [53]. To trigger immune responses against the other 3 serotypes, DENV1, DENV3, and DENV4, the coding sequences of DENV2 PDK-53 were replaced with those of DENV1, DENV3, and DENV4, which encode the prM and E proteins. The vaccine is then formulated by transfecting recombinant RNAs in Vero cells [37]. The inclusion of the entire backbone of the DENV2 virus gives TAK-003 a comparative replication advantage and reducing the DENV2 component by 1 logarithm unit also helps the vaccine trigger a more balanced immune response compared with Dengvaxia [54]. Another advantage of the PDK-52 backbone is that it enables the vaccine to induce DENV-specific cellular and humoral responses simultaneously.

Up to December 1st, 2021, Takeda has conducted 5 phase III trials for this vaccine, the largest of which was the DEN-301 trial, also known as TIDES [55]. TIDES involved 20,099 children aged 4 to 16 years in 8 countries, a sample size rarely seen in other vaccine studies [55]. Moreover, in previous phase I and II trials in Columbia and other dengue prevalent countries, TAK-003 successfully induced immune responses against all 4 dengue serotypes in children and adolescents aged 1.5 to 11 years [56]. Compared with its attenuated chimeric predecessor, TAK-003 is suitable for seropositive and seronegative patients, significantly reduces the risk of hospitalization related to dengue fever, protects children aged below 9 years, and requires only 1 dose to induce cellular and humoral immune responses. According to previously published data, TAK-003 “elicited antibody responses against all 4 serotypes, which persisted to 48 months post-vaccination, regardless of baseline serostatus” [57]. Even though the TAK-003 vaccine is not currently licensed anywhere in the world, we think it will be soon according to the current clinical data.

### 3.2 Genetic vaccines

As the COVID-19 pandemic prevails globally, a “new” type of vaccine has been developed. Genetic vaccines, such as DNA and mRNA vaccines, utilize part of the virus’ genes to formulate immune responses instead of using recombinant bacteria or viruses as in the traditional vaccines [58]. Although similar technology was studied for other viruses in the 1990s, it was not until the current pandemic that these vaccines were developed further. The success of the mRNA vaccine strategy against COVID-19 has also encouraged scientists to adopt similar techniques for previously unsolved health problems, such as dengue.

DNA vaccines involve direct viral genetic information in a plasmid form, containing several virus-specific antigens encoded by genes [59]. Several DNA vaccine studies of dengue have been proposed. Two decades ago, scientists fused immuno-stimulatory CpG DNA motifs with a DNA vaccine expressing DENV2 prM/E proteins and showed satisfactory immune protection against DENV2 [60]. However, weak immunogenicity is still the most significant problem for DNA vaccines. A more recent study boosted the immunogenicity of DNA vaccines against DENV by including antigens that targeted dendritic cells (DCs), the primary antigen-presenting cell (APC) of the immune system [61]. Because DCs connect innate immunity and adaptive immunity, this vaccine can induce cellular and humoral immune responses. The targeting effect is formed by fusing the antigen with a single-chain Fv antibody (scFv) specific for the DC endocytic receptor DEC205 [61]. Compared with mRNA counterparts, DNA vaccines are more stable, cheaper, and thus more suitable for mass production. However, its disadvantages include a lack of immunogenicity, making the mRNA vaccine more popular in current scientific research [37].

Another representative genetic vaccine is the mRNA vaccine. Instead of containing a DNA plasmid, mRNA vaccines use mRNAs that encode viral proteins, sacrificing stability for effectiveness. In May 2021, a serotype-specific nucleotide-modified mRNA vaccine for DENV1, encoding its prM and E proteins, was developed [62]. Similar to other mRNA vaccines, the candidate vaccine was encapsulated in lipid nanoparticles (mRNA-LNP) to protect it from the host's enzymes [63]. This vaccine triggered sufficient numbers of antigen-specific antibodies and evoked robust T cell immunity, while achieving minimal serum cross-reactivity and reduced ADE [62].

Unfortunately, several innate properties of the virus have hindered the development of a well-rounded vaccine with high effectiveness and low risk. Continual effort is required to develop a more general and acceptable DENV vaccine.

## 4 Humoral immune responses to DENV infection

Humoral immunity is also known as antibody-mediated immunity. During DENV infection, neutralizing antibodies target the prM, E, and NS1 proteins. Following primary infection (1°) or vaccination, B cells respond after innate immunity and continuously produce B cell receptors (BCRs) that further differentiate into virus-specific antibodies. Among all immunoglobulins, IgM antibodies are differentiated and detectable in the blood of 50% of patients within 3–5 days of disease onset and in approximately 99% of patients within 10 days. IgM antibody levels peak approximately 2 weeks after onset and decrease to an undetectable level at approximately 2–3 months.

In contrast, IgG antibody levels remain low for approximately 1 week, but then increase continuously afterward. Thus, the levels are detectable for months or even years after the patient has recovered, providing long-term protection against a particular DENV serotype [64]. Furthermore, when a secondary (2°) infection occurs, the level of IgG antibodies targeting the primary infection remains high, whereas the IgM level gradually increases. Therefore, an IgG/IgM ratio of 1.10 is commonly used as a clinical indicator to determine whether a patient has a secondary infection [65].

In addition to serving as a diagnostic indicator in the early stage of disease, the most critical function of antibodies is their neutralizing ability to defend against viral infection. The antibody-mediated neutralization of flaviviruses follows the “multiple hit theory”. Namely, a critical number of antibodies must bind to a single virus to neutralize it, but currently, no study has yet discovered this critical threshold value for DENV [66], [67]. Therefore, enhancing our understanding of the neutralization ability of DENV antibodies is crucial for vaccine development. In this review, the effects of 3 important factors on the neutralization of DENV by antibodies are discussed.

First, the accessibility of epitopes determines whether epitopes on the antigen interact with antibodies to trigger an immune response. This accessibility is influenced by the heterogeneity and surface conformational dynamics of viruses. The structural instability of DENV at various pH and temperature levels is termed “viral breathing”, which regulates the accessibility of epitopes and thus affects the neutralization of antibodies. After prolonged or high-temperature incubation, the binding and neutralizing abilities of antibodies were improved [68]. Under the influence of viral breathing, the previously hidden epitopes of EDIII were exposed, which allowed the highly neutralizing 1A1D-2 mAb to bind and inhibit the attachment of dengue virions [69].

**Fig. 1:**
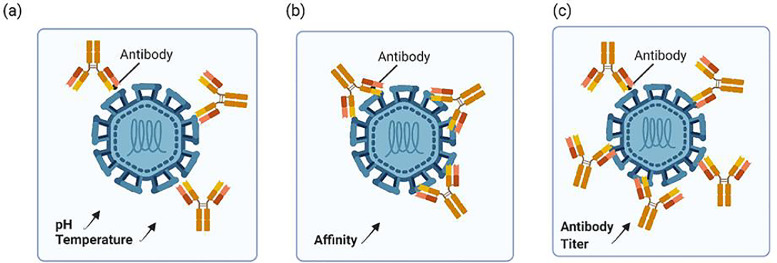
The neutralizing capacity of DENV-specific antibodies can be determined by 3 factors: (a) The accessibility of epitopes: influenced by the heterogeneity and surface conformational dynamics of viruses. When incubated in a relatively high temperature or relatively high pH environment (weak basic), the neutralizing capacity is enhanced. (b)The affinity and avidity: when the affinity (affinity and avidity) increases, the neutralizing effect is also increased (c) Antibody titer: high antibody titer is correlated with higher neutralizing capacity.

Second, the affinity and avidity of antibodies collectively determine how many antibodies bind to an antigen at a particular concentration. Affinity indicates the binding strength at a single antigen-binding site, whereas avidity indicates the total binding strength between a polyvalent antibody and several epitopes. Overall, affinity maturation is the critical factor for generating antibodies with strong affinity. Antibodies induced by natural infection or vaccine injection induce somatic mutations in the germinal centers of lymphoid organs. B cell receptors with a point mutation in heavy or light chains obtain a greater binding strength toward foreign antigens. After several cycles of mutation, propagation, and natural selection, antibodies with the highest affinity are retained and the affinity or avidity of polyclonal antibodies is increased [70]. Affinity maturation optimizes the neutralizing ability of the antibody and enhances antibody-dependent cellular cytotoxicity (ADCC). Therefore, measuring the affinity maturity of a vaccine candidate will help identify a more effective dengue vaccine.

Third, the antibody titer is also an essential factor that contributes to antibody neutralization, because it reflects the patient's serum antibody levels. Within a specific titer range, the neutralizing antibody induced by DENV infection fails to provide immune protection and increases the risk of acquiring severe dengue fever following a secondary (2°) infection. Furthermore, as mentioned above, Dengvaxia has a high risk of severe dengue fever and hospitalization in serologically negative recipients [38]. A previous study confirmed that the disease severity of 2° DENV infection correlated with preexisting antibody levels. In that study, ≤1:40 antibody titers were associated with a 7.4-fold increased risk of DHF (compared with serologically negative individuals) [71]. Based on these studies, severe dengue is clearly not as strongly associated with higher or lower antibody levels as it is with antibody titers. Given the possible risk of severe dengue, the strict monitoring of neutralizing antibody titers induced by vaccine candidates might help avoid postvaccination harm.

### Antibody-dependent enhancement (ADE) in dengue

When developing DENV vaccines it is important to study antibody-dependent enhancement (ADE). ADE is considered a possible mechanism explaining the increased disease severity after a secondary heterotypic DENV infection [12], which is typically associated with an increase in serum viremia [72]. For example, antibodies enhance the DENV 2 infection capacity at specific titers. Another study reported that newborns with waning maternal antibody titers also had a higher risk of DHF/DSS [73], [74]. After infection, antibodies in the serum are mainly composed of IgG with different affinities and neutralization capacities (Fig. 1). In addition to the desired serotype-specific (TS) and protective antibody responses, DENV infection might also induce the production of non-specific non-neutralizing or weakly neutralizing cross-reactive (CR) antibodies [12]. However, studies showed that both types of antibodies protected individuals from severe illness caused by secondary exposure over a specific interval. An experiment conducted in Nicaragua showed that CR antibodies that persisted at a high titer after primary infection (1°) reduced the risk of severe illness related to a secondary (2°) infection [75].

Two other experiments confirmed that the shorter the interval between 2 DENV infections (< 2 years), the more likely the CR antibody can provide cross-protection against heteromorphic infections [76], [77]. The ADE phenomenon, which provides protection in the short term and leads to severe dengue fever in the long term, is probably caused by the natural decay and low binding affinity of the CR antibody. Because the half-life of a DENV antibody is approximately 4 years [78], the longer the interval between 2 infections, the lower the level of CR antibodies with weak neutralization ability is expected. Therefore, when binding to the virus, these antibodies fail to achieve neutralization and bind to the Fcγ receptor of immune cells, such as monocytes and macrophages, through their Fc segment, leading to the increased entry of DENV and increased severity of dengue fever [79], [80].

The ADE phenomenon is composed of exogenous and endogenous components. Exogenous ADE increases the number of infected host cells, as mentioned above. Endogenous ADE induces host cells to produce more DENV virions by inhibiting the production of type 1 interferons (IFN-I) and interleukin-10 (IL-10) [81]. Although 15 times higher risk of severe dengue was observed in children comparing with than in adults [82], Dengvaxia has not been approved for use in children aged less than 9 years. Similarly, all other vaccine candidates must overcome the ADE problem before being widely used in children and serologically negative populations.

## 5 Cellular immune response to DENV infection

Because of ADE, scientists are now shifting their focus from humoral immunity to cellular immunity. T cells are the main component of cellular immunity and are further classified into CD4^+^ and CD8^+^ T cells. CD8^+^ T cells, also known as cytotoxic T lymphocytes (CTLs), directly kill infected cells or help recruit cytokines (ie, IFN-γ and TNF-α) to control viral propagation. However, the function of CD4^+^ T cells (also known as T helper cells) is to enhance the immune response mediated by B cells and CD8^+^ T cells and transmit signals back to innate immune cells to produce inflammatory and antiviral cytokines and promote cytotoxicity and immune memory [83].

**Fig. 2:**
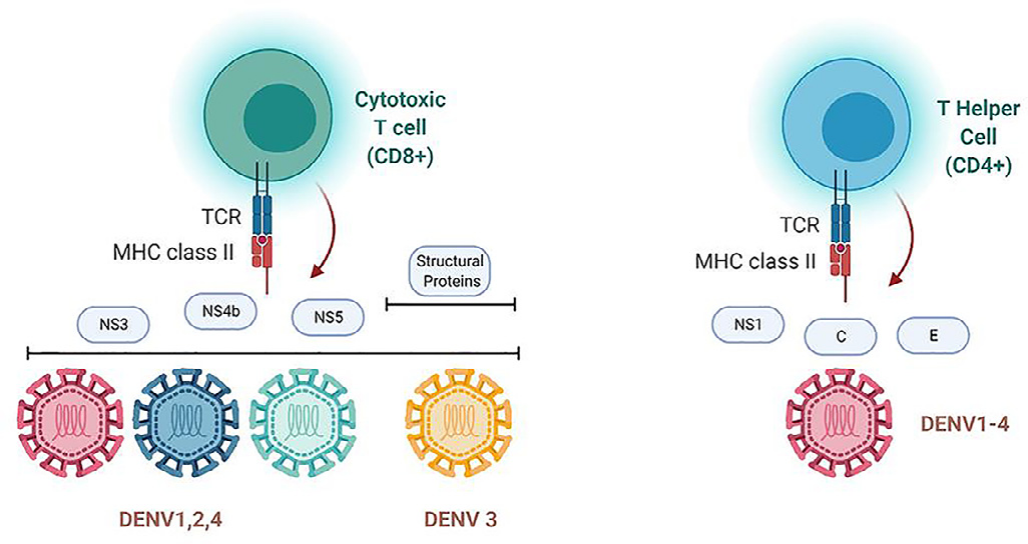
The cellular immune response of DENV is mainly determined by cytotoxic (CD8^+^) T cells and T helper cells (CD4^+^). CD8^+^ T cells mainly recognize nonstructural proteins such as NS3, NS4b, and NS5 from DEV1, 2, and 4. During DENV3 infection, CD8^+^ T cells recognize structural and non-structural proteins. CD4^+^ T cells recognize C and N structural proteins and non-structural proteins such as NS1.

DENV antibodies primarily recognize structural protein epitopes with high immunogenicity, whereas the cellular response is triggered by various structural and nonstructural proteins. CD8^+^ T cells predominantly bind to the immunodominant epitopes of each DENV serotype. During infection with DENV1, DENV2, and DENV4, the CTLs mainly recognize nonstructural proteins such as NS3, NS4b, and NS5, whereas during DENV3 infection, they simultaneously identify structural and nonstructural proteins [84]. CD4^+^ T cells recognize antigen determinants located on structural proteins C and E and the nonstructural protein NS1 (Fig. 2) [85]. To date, the protective role of T cells has been well established in animal models and human trials. An experiment using IFN-α/βR/mice showed that mice lacking CD8^+^ T cells had a higher viral load than controls, but the use of dominant epitopes to induce cellular responses promotes virus clearance [86]. Moreover, another study using BALB/C mice confirmed that mice do not survive a lethal challenge with DENV2 in the absence of T cells, despite a high level of neutralizing antibodies [87]. Because CTLs reduce the risk of severe dengue caused by ADE [88], cellular immunity has become a high-profile topic in dengue vaccine development in recent years.

In contrast to CD8^+^ T cells, CD4^+^ T cell deficiency has no direct effect on the viral load, DENV-specific IgG/IgM titers, or antibody neutralization capacity. However, immunization with T helper cell (Th)-targeted epitopes significantly reduced the viral load [89].

The protective role of cellular immunity observed in animal models has also been validated in humans. A recent study examined the differences between asymptomatic infections and children with clinical symptoms. The study reported a significant increase in T cell-mediated apoptosis in the former group, which promoted DENV clearance [90]. Another study focused on the difference between T cells from patients with mild or severe syndromes. These T cells, specifically targeting NS3 and NS5, produced significantly higher amounts of interferon-γ (IFN-γ) and tumor necrosis factor-α (TNF-α) in patients with a mild illness, suggesting that the immune response mediated by T cells potentially influences disease severity [91]. In summary, an effective immune response against DENV must consist of neutralizing antibodies and T cells. The absence of either would constitute a significant breach in the host's protective mechanisms.

At present, no definite link between T cells and immune protection has been established, although the factors associated with cellular responses still reflect their protective functions. Therefore, the interaction between specific factors and T cell-mediated immune responses or pathogenicity must be clarified to evaluate future vaccine effectiveness.

## 6 Vaccines and efficacy

Although currently available vaccines and those in clinical development successfully induce antibody or T cell responses, no vaccines are currently widely available because of several factors. We discuss 3 factors that potentially affect vaccine efficacy, and Dengvaxia is used as an example to illustrate the importance of these factors.

The first factor is the baseline serological status and age of the recipient. Since its introduction, the Dengvaxia vaccine has remained controversial because its effectiveness is primarily influenced by the baseline serological status of the recipient (ie, prior DENV infection) and age, creating an unbridgeable impediment for large-scale vaccination. A retrospective study recently revealed a 3.06% hospitalization rate in serologically negative people aged 2 to 16 years caused by DENV infection within 5 years of Dengvaxia vaccination [38]. For the placebo group, the hospitalization rate was 1.87% and decreased with increasing age [38]. This study showed that Dengvaxia does not provide adequate protection for young people with no previous exposure to DENV. Because older adults living in DENV-endemic areas are more likely to have a baseline seropositive status and more well-rounded immunization protection through vaccination, the inoculation of Dengvaxia is restricted to nonpregnant people over the age of 9 years in high-risk areas.

The second factor that affects vaccine effectiveness is genetic differences in the dengue strain. Dengvaxia and other vaccine candidates have shown significant differences in efficacy among the 4 DENV serotypes, with the best efficacy against DENV4 and another vaccine, TAK003, showing the best efficacy against DENV2. In addition to the difference in efficacy, an approximately 6% difference within a single serotype has been reported [8]. Several studies have suggested that differences in the genotypes of DENV1–4 serotypes might lead to variations in the epitopes in the prM and E proteins, thereby affecting the neutralization ability of antibodies induced by natural infection or vaccination [[9], [10],[92], [93]]. Scientists conducted a comparative analysis of cyD14/15 phase III clinical trials to understand whether the serotype difference affected the efficacy of Dengvaxia. The retrospective results revealed that the vaccine's efficacy negatively correlated with the degree of variation in amino acid sequence between the viral strain used to produce the vaccine and the endemic viral strain [94]. However, genetic differences were most likely to affect vaccinated individuals younger than 9 years, and thus, the vaccine-induced cellular immune response is not ideal.

Last, the comprehensiveness of the vaccine-induced immune response also plays a role in vaccine efficacy. For example, 1 explanation for the poor efficacy of Dengvaxia is that it lacks an antigenic determinant (a nonstructural protein of DENV) capable of inducing a T cell-mediated immune response. Humoral immunity alone is unable to provide complete protection and increases the risk of DHF/DSS because of antibody-dependent enhancement.

## 7 Concluding remarks

The annual cases of dengue fever have increased continuously during the last several decades. If countries ignore the risk and relax vigilance on preventing and controlling dengue, future seasonal outbreaks will ultimately worsen the already overburdened medical system, especially in the context of the COVID-19 pandemic. Therefore, the development of human immunization and vaccine strategies for DENV is still a topic of great concern. In this article, we introduced dengue virus in detail and analyzed its interactions with humoral and cellular immunity. The article also uses the only licensed vaccine Dengvaxia as an example to illustrate the efficacy and potential problems associated with the current vaccines. For future candidate vaccines, stronger cellular immune responses should be induced instead of determining how to avoid ADE. Therefore, the next generation of DENV vaccines for wide use in humans must combine high titers of antibodies and activated T cells to balance the immune protection against the 4 serotypes and avoid ADE. One possible immune strategy is to use the NS1 antigen, which can induce effective antibody responses, and unlike E or prM structural proteins, does not lead to ADE [95]. Given the limited access to Dengvaxia vaccination and the practical challenges of serum testing, next-generation dengue vaccines should be more effective, safe, and easy to administer.

## Declaration of competing interest

The author(s) declared no potential conflicts of interest with respect to the research, authorship, and/or publication of this article.

## Ethical statement

We hereby, assure that our research strictly adheres to all the ethical guidelines in concern.
